# mCAF: a multi-dimensional clustering algorithm for friends of social network services

**DOI:** 10.1186/s40064-016-2420-1

**Published:** 2016-06-17

**Authors:** Hsien-Tsung Chang, Yu-Wen Li, Nilamadhab Mishra

**Affiliations:** Department of Computer Science and Information Engineering, Chang Gung University, Taoyuan, Taiwan

**Keywords:** Clustering algorithm, mCAF, Friends clustering, Social network services

## Abstract

In recent years, social network services have grown rapidly. The number of friends of each user using social network services has also increased significantly and is so large that clustering and managing these friends has become difficult. In this paper, we propose an algorithm called mCAF that automatically clusters friends. Additionally, we propose methods that define the distance between different friends based on different sets of measurements. Our proposed mCAF algorithm attempts to reduce the effort and time required for users to manage their friends in social network services. The proposed algorithm could be more flexible and convenient by implementing different privacy settings for different groups of friends. According to our experimental results, we find that the improved ratios between mCAF and SCAN are 35.8 % in similarity and 84.9 % in *F*_1_ score.

## Background

With the recent implementation of Web 2.0, an increasing number of users are posting personal information, moods, and life events to the Internet through instant messaging software, blogs, and social network services to share their lives with family and friends. This sharing has become an indispensable part of many people’s lives.

Facebook is a social network service that was founded in 2005. Based on the findings of a 2006 survey at a university in the United States, the average number of Facebook friends of each student was 272 (Matthew Robert 2006). In 2008, students at another American university had an average of 246 Facebook friends (Walther et al. 2008). A study (Wilson et al. 2012) in 2012 showed that approximately one-fifth of Facebook users had less than 25 friends, and half of users had more than 100 Facebook friends; the global average at that time was 130, much lower than the average of 214 in the United States. Based on 2014 statistics, Facebook has nearly 1.3 billion users and remains “the world’s most widely used social networking service”.

In recent years, together with the growing popularity of mobile devices, the number of Facebook users has continued to increase. A single Facebook user with hundreds or even thousands of Facebook friends has become an extremely common phenomenon, which exceeds a user’s capacity to manage their list of friends (up to 150 friends) (Dunbar 1993). The increasing number of friends markedly reduces a user’s willingness to cluster friends individually (Simon Jones 2010). People tend to cluster friends only when they want to open a chat window to chat with specific friends, send a group message, or filter uninteresting messages. Several problems arise because friends on Facebook contain various types of people, including family, friends, friends of friends, and colleagues. If a user’s funny pictures or words are seen by relatives or family elders who do not agree with or even disapprove of the posted content, it may affect their perceptions and views towards the user. Additionally, frequent uploads of photos that show a user drinking alcohol and dancing in nightclubs and bars may give co-workers and supervisors negative impressions of the user.

Reference (Kelley et al. 2011) reported that instead of uploading such information and setting privacy controls, only a specific type of information is disclosed to a specific group of friends. Additionally, most people prefer not to upload this type of sensitive information to Facebook at all. One reason for this preference is that a user’s number of friends is too large, and only a few users are willing to take the time to sort their friends. Another reason is that the Internet and mobile devices are extremely popular, and the transfer of information is easy, which allows people to gradually ignore the concept of maintaining privacy. Setting different privacy privileges is not sufficient to motivate a user to cluster friends. Thus, we designed an automatic clustering algorithm in this study that clusters and groups friends using a clustering concept given different privacy settings for different clusters; this algorithm could thereby prevent certain friends from viewing inappropriate information.

Clustering is often used when analyzing large amounts of data. Because it is impossible to know in advance how many categories the subjects will be divided into, the proposed clustering concept uses “distance” as the basis for clustering; clustering treats subjects with closer “relative distance” as subjects with higher “similarity” and then categorizes them into the same group. The basic clustering algorithm and several accelerated algorithms have been proposed (Murtagh 1983). As reported in one study (Xu et al. 2007), the structural clustering algorithm for networks (SCAN) defines two special user roles in the Internet, hub and outlier. We use these two special roles to allow users to decide which cluster they belong to such that the error rates of clustering are reduced. Reference (Hossmann et al. 2012) also describes a multi-dimensional network analysis via structural analytics that considers certain dimensions, such as social meetings, communications, and mobility; the datasets that are considered in that study contain social, mobility, and communication information.

In this study, we define four types of measurements for group friends: social circles, regions, organizations, and tie strength. The social-circle represents separating social factions, common friends list, and messaging interactions within a Facebook community. The regions measurement uses regional locations to define friend types based on the distances between users’ hometowns or current locations among friends. The organizations measurement clusters friends based on affiliations using schools attended or companies worked for. The tie-strength measurement represents the degree of interaction between two friends on social networks, which is calculated by the social-degree parameter (Tsai et al. 2014).

In real social networks, there are different levels of relevance between people, which can be described by different weights (i.e., weight or degree of association) in a network; this network structure can be used for analysis. In this paper, we consider both this weight and the network structure. We propose a new algorithm based on the SCAN algorithm called the Multi-dimensional Clustering Algorithm for Friends (mCAF), which defines weight values using the measurements discussed above. Using data from the Facebook API, clustering was first performed, and the similarity between the results was compared with the experimental subjects’ card-sorting (Kelley et al. 2011) results. The goal is to ensure that the mCAF algorithm clusters a user’s friends as similarly as possible to the way the user would cluster their friends.

The following sections are structured as follows. Section “Related works” summarizes the literature that has been published in recent years, including studies of basic clustering algorithms and the SCAN algorithm. Section “mCAF: multi-dimensional clustering algorithm for friends” defines the proposed measurements and the mCAF algorithm. Fourth section presents the results of the experiment, and final section presents the conclusions.

## Related works

Network clustering is a method commonly used to analyze the structure and characteristics of social networks. There are two types of hierarchical clustering methods: agglomerative and divisive. Agglomerative hierarchical clustering can be best used as a tagging system in large social networks (Shepitsen et al. 2008). Divisive hierarchical clustering repeatedly divides a given cluster into smaller clusters and analyzes the edges connecting vertices in the same cluster (Costa et al. 2016). In agglomerative hierarchical clustering, there are many ways to define distance (e.g., single, complete, and average linkages).

A previous study (Murtagh 1983) defined the nearest-neighbor (*NN*) graph as a collection of points, where *NN* (*p*) represents the nearest neighbors from point p. If two points, p and q, satisfy *NN* (*p*) = *q* and *NN* (*q*) = *p*, then points p and q are defined as reciprocal *NN*s (*RNN*s). An *NN*-chain is a chain composed of *NN* (*p*), beginning from an arbitrary point and ending at *RNN*s. A previous paper used the aforementioned definitions and proposed four fast algorithms. The first algorithm finds all *RNN* pairs and connects those that are closest to each other. The second and third algorithms find an *NN*-chain and then connect the closest points, subsequently creating the *NN*-chain. The fourth algorithm finds all *NN*-chains and then sequentially connects the nearest two *NN*-chains.

**Fig. 1:**
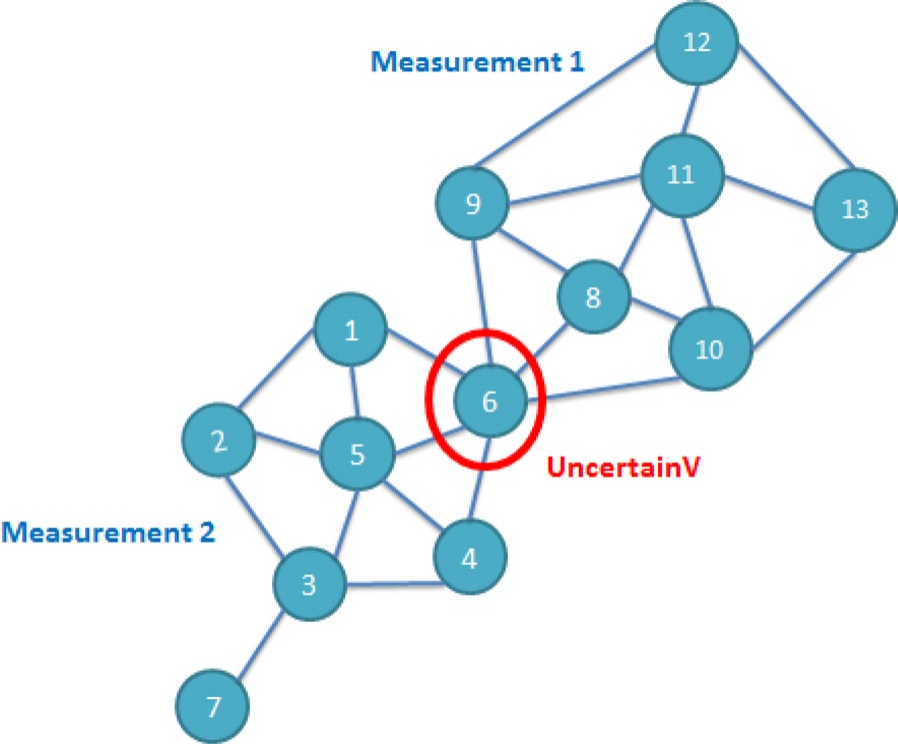
Network with 2 clusters, a hub and an outlier

Another clustering algorithm is called the SCAN algorithm (Xu et al. 2007), which considers the structural differences of a network diagram to perform clustering and defines two special roles: a hub, which connects two or more clusters that are highly associated, and an outlier, which has a relatively lower level of association with other members in a cluster. Figure 1 shows an example that has two clusters. In this example, node 6 is a hub, node 7 is an outlier, and the remaining nodes are members of one of the two clusters.

The automated friends clustering or grouping algorithms used for online social networks are discussed in reference (Eslami et al. 2014). In that study (Eslami et al. 2014), the researchers propose that manual clustering of large numbers of friends overburdens social network users; thus, interested social network users may use automatic clustering algorithms to create quick groupings of their large numbers of social-network friends with minimal effort. Integrated interfaces are suggested to allow Facebook users to modify these groupings or friend clusters per their requirements and convenience. One of the most popular tools, which is called the Facebook smart list, has also been proposed to address this problem (Gao et al. 2012). The Facebook smart list is a recommendation-based mechanism that can be effectively used by Facebook users to automatically group their friends.

In an experiment (Simon Jones 2010) that sought to determine the factors considered by experimental subjects when clustering friends, the authors collected information corresponding to all Facebook friends of 15 subjects and asked the subjects to cluster friends using a card-sorting (Kelley et al. 2011) method. The subjects answered several questions before the experiment. Using the two aforementioned methods, the authors summarized the size measurement that can be used for the clustering of users’ friends: social circles and cliques, tie strength, temporal episodes, geographical locations, functional roles, and organizational boundaries. Among these factors, the most commonly used measurement was social circles and cliques, followed by tie strength. After the card-sorting task was completed, we used the SCAN algorithm to cluster the data from Facebook. Finally, we compared the similarity of the results provided by the card-sorting method and the SCAN method (1). First, we defined the card-sorting clustering results as set *C* = {*C*_1_*, C*_2_*, C*_3_*,…, C*_*m*_} and the SCAN clustering results as set *G* = {*G*_1_*, G*_2_*, G*_3_*,…, G*_*n*_}.

The similarity between *C*_*i*_ (*1* ≦ *i* ≦ *m*) and *G*_*j*_ (1 ≦ *j* ≦ *n*) is represented by *S*_*ij*_. This method calculates a similarity percentage score as the number of friends in both groups (*C*_*i*_ intersect *G*_*j*_) divided by the sum of distinct members of different groups (*C*_*i*_ union *G*_*j*_). The similarity values of the experimental results ranged between 18.1 and 79.5 % with an average of 44.8 %. Because this method only considers the structure of the network, its accuracy remains inadequate; therefore, we concluded that this method is unsuitable:

1 $$Sim\left( {C,G} \right) = \frac{{\mathop \sum \nolimits_{i \le m, j \le n} S_{ij} }}{{Max\left( {m, n} \right)}}$$

This experiment also showed that when subjects classified Facebook friends, several of their friends caused anxiety such that the subjects did not know how to cluster the friend. Most of these people were identified by the SCAN (Xu et al. 2007) algorithm as hubs or outliers. We suggested that the subjects mark these types of people so that they could be removed from the algorithm-based clustering process and only be used by the subjects for manual clustering.

Several studies have explored weighted network analyses (Barrat et al. 2004; Phan Binh and Fjeldstad Øystein 2013; Tore Opsahl 2009) and defined open triplets, which are composed of two edges, and closed triplets, which are composed of three edges. Additionally, a triangle is defined as containing three closed triplets. There are many ways to calculate the weight of each triplet, including the arithmetic mean, geometric mean, maximum, and minimum; however, most researchers use the geometric mean. The weighted clustering coefficient (2) is defined as:

2 $$C_{\omega } = \frac{Total\, Value \,of \,Closed \,Triplets}{Total \,Value \,of \,Triplets} = \frac{{\mathop \sum \nolimits_{{\tau\Delta }} \omega }}{{\mathop \sum \nolimits_{\tau } \omega }}$$

The Group Recommendation System (GRS) (Baatarjav et al. 2008) provides a calculation method and suggests that the users join school clubs similar to their own characteristics. The GRS uses 15 features that are normalized to values between 0 and 1 as the bases for calculating the distance (i.e., weight) between people, including time zone, age, etc.

Multi-dimensional clustering algorithms on social networks are progressively gaining popularity due to the information and insights produced using large-scale social data. The user’s opinions, comments, and likes in social media have significant relationships with the popularity of that post (Tan et al. 2014). The users of social media platforms such as Facebook often like several brands, which can be clustered into several groups and then analyzed. Reference (Wallace et al. 2014) describes multi-dimensional cluster analysis as a strategy for identifying different Facebook users’ fan groups and provides insights to prompt further research analytics. Reference (Mcauley and Leskovec 2014) considers both network structures and profile information while analyzing a user’s clusters on social networks.

## mCAF: multi-dimensional clustering algorithm for friends

The users of social media websites often upload several articles or messages and do not consider who will see the information, while other users simply choose not to upload any private messages. It is thus good practice to set different levels of privacy for different groups of friends or people to allow for easy use, reduced concern, and increased protection of user privacy.

**Fig. 2:**
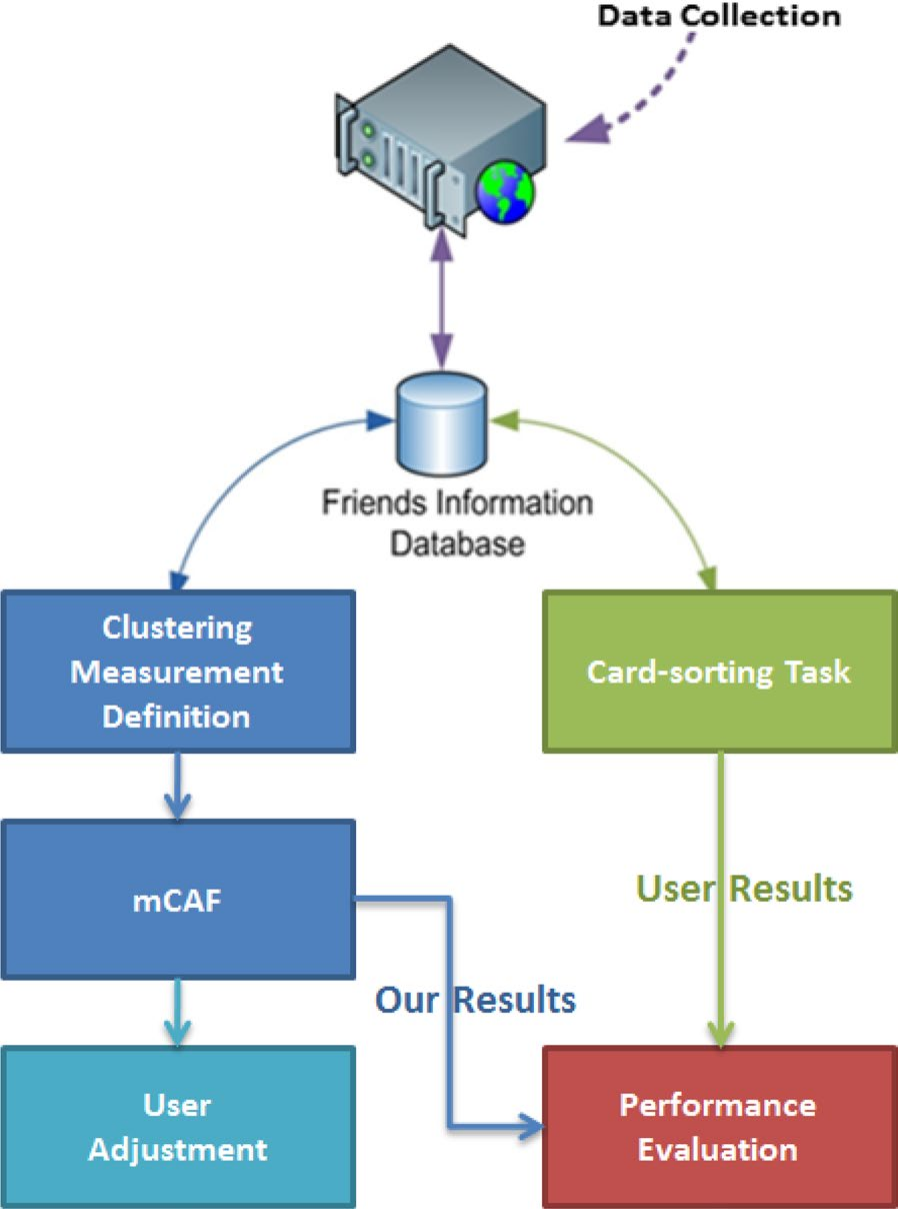
Basic architecture of the proposed method

This section introduces the proposed friend-clustering algorithm called mCAF. Figure 2 shows the proposed approach and framework, which consists of the following six steps.

### Data collection

The amount of social-network data is currently growing exponentially. Thus, many researchers are investigating different data collection frameworks for data elicitations and analysis. Reference (van Dam and van de Velden 2015) describes a data-collection framework that can be used to explore user profiles and identify segments based on these profiles. In that study, the authors visualize how data from Facebook can be operationalized to obtain insights into a given user connected to the Facebook social network. Online data collection is complex, and in certain scenarios, manual data collection mechanisms are preferred due to the lack of adequate technological mechanisms (Aggarwal 2015). In this study, data are collected from the social network service Facebook, which is currently the world’s most widely used social network service. We used the Facebook Graph API (Version 2.0, 2014) to retrieve user information. There are several limitations when using the Graph API; for example, no more than 600 directives can be obtained within 600 s, and there is a limited time to retrieve data of interest. We thus issued several Graph API requests to retrieve all the data required for the proposed algorithm.

### Clustering measurements definition

Based on the measurements described in reference (Simon Jones 2010) and information that could be retrieved from Facebook, we defined four measurements for use by the mCAF algorithm as follows: social circles, regions, organizations, and tie strength.

#### Social circles

Social circles are common criteria that have been used to cluster friends in past studies. A social circle is a group of people who have the same interests or join the same activity. We thus define *M*_*ij*_ as the number of mutual friends of user *i* and *j* and *G*_*ij*_ as the interaction value in Facebook group functions. We also define *C*_*k*_ as a collection of article identification numbers. For example, *C*_*k*_ = {1,2} indicates that the *k*th friend of one subject leaves messages after articles 1 and 2 have been posted by the subject. A subject is considered to have *n* friends on Facebook. Thus, we quantify the subject’s interactions within the community to obtain *S*_*ij*_ with Eq. (3) and then normalize the results (5):

3 $$G_{ij} = |C_{i} \cap C_{j} |$$

4 $$MG_{ij} = M_{ij} + G_{ij}, \quad MG = \{ MG_{xy} |x,y \in 1,2,3 \ldots n\}$$

5 $$S_{ij} = \frac{{MG_{ij} }}{{Max\left( {MG} \right)}}, \quad S = \left\{ {S_{xy} |x,y \in 1,2,3 \ldots n} \right\}$$

#### Regions

Regions are based on location information from Facebook. Certain friends live in the same geographic location (e.g., neighbors and fellow students). Users’ hometowns and current locations of residence can be obtained from Facebook.

We first determine the latitude and longitude of the hometowns and locations of the subjects and their Facebook friends. We then calculate the distances between them and store them as a dataset described by {*D1, D2, D3, D4*}. For example, to calculate the distance between *A* and *B*, *D*_1_ represents the distance between the hometowns of *A* and *B*; *D*_2_ represents the distance between the current residences of *A* and *B*; *D*_3_ represents the distance between *A*’s hometown and *B*’s current residence; and *D*_4_ represents the distance between *A*’s current residence and *B*’s hometown. We calculate *R*_*ij*_ as shown in Eq. (6):

6 $$R_{ij} = \alpha \times D_{1} + \beta \times D_{2} + \gamma \times D_{3} + \delta \times D_{4}$$

#### Organizations

If two people attended the same school or worked in the same company, they have a connection, and the organizations measurement is set equal to 1. If no connection is present, the organizations measurement is set equal to 0. We define *O*_*ij*_ to store this value.

#### Tie strength

In certain instances, we will cluster some best friends as one group so that they can share private or important events. Conversely, we have some unfamiliar friends who are still kept as friends only because users may feel embarrassed about removing them. Thus, users may choose to cluster those unfamiliar friends into one group. We retrieve related information from Facebook and use the method described in Tsai et al. (2014) to calculate the tie strength as *T*_*j*_, which indicates the tie strength between a user and their *j*th friend.

### Multi-dimensional Clustering Algorithm for Friends (mCAF)

There are two types of clustering methods that have been proposed by researchers to date. One type of method (i.e., the SCAN method) uses the composition of the structure of an entire network (Xu et al. 2007). The other type of method only considers the weights of the edges (Barrat et al. 2004; Phan Binh and Fjeldstad Øystein 2013; Tore Opsahl 2009). We believe that both factors are important to cluster friends; thus, the proposed approach in this study combines these two concepts. We treat the subject and their friends as vertices on a graph, and the connections between friends are treated as edges. The values defined by the different measurements are the weights of the edges.

We first select the best measurement from social circles, regions, and organizations to cluster each node (i.e., friend). Although we proposed a multi-dimensional clustering algorithm, we will cluster friends into one group according to only one measurement. For examples, one group is clustered according to organizations measurement and another is clustered according to regions measurement. Each friend is connected to others by the measurements we proposed, and we need to decide which measurement is the best one for this friend to be clustered with others. We try to count the number of each measurement with the highest value within the edge. The measurement with the highest number of count will be treated as the best measurement to this friend. We also can identify uncertain nodes, which do not have the best measurement.

We start from any node *p* and, in turn, connect adjacent nodes if they are similar to node *p* and have the same best measurement as that of node *p* until all nodes are clustered or the similarity is not sufficiently high for any group. Finally, we use the tie strength to choose the top *n* (*n* is set to 15) closest friends to form a group of close friends if those friends are scattered among various groups. Conversely, we do not deliberately separate them to create a new group if those close friends are already clustered into only one or two groups.

**Fig. 3:**
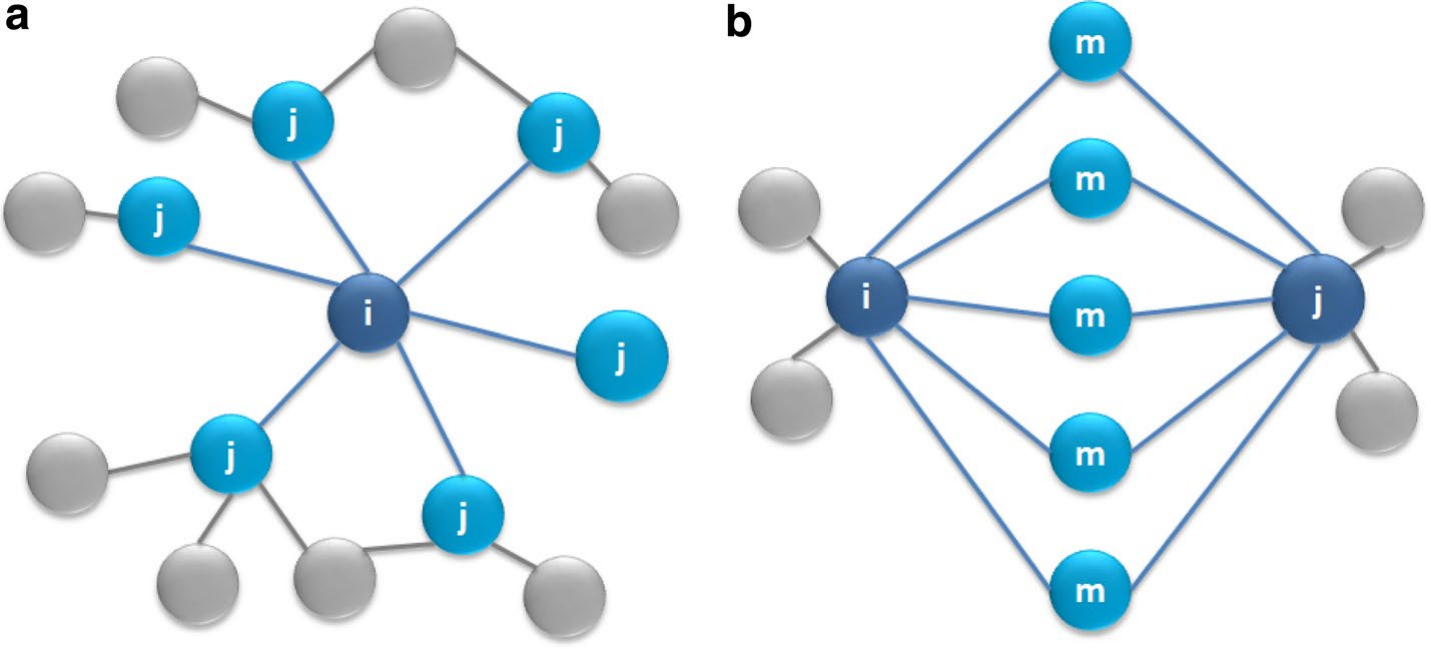
Example of nodes in a graph. **a** Edges between i and j. **b** Mutual connection edges of i and j

In this paper, we map a user’s friends into un-directed, weighted graphs. We now formally describe the proposed method mCAF. We define the entire graph as *G* = {*V, E*}, in which *V* is the set of vertices and *E* is the set of edges, defined as $\left\{ { E_{i,j} \left( {e_{i,j}^{k} } \right) } \right\}$, which represents a connection if a value $e_{i,j}^{k}$ is greater than zero between nodes *i* and *j* under measurement *k* (*k* = 1–3). As shown in Fig. 3a, there exist 5 edges connected to a set of *j* where one $e_{i,j}^{k}$ value is greater than zero. We now further define the vertex structure and structural similarity and then describe the pseudo code of the proposed mCAF algorithm.

#### Definition of vertex structure

Let vertex *i* *∈* *V*, where the structure of *i* is defined by its neighborhood, denoted by *Γ*(*i*) in Eq. (7):

7 $$\varGamma \left( i \right) = {\text{\{ }} j | j \in V \wedge E_{i,j} \in E \}$$

#### Definition of the weight summary of one measurement to one node

Equation (8) defines the summary values of measurements from vertex *j*, which is connected to *i*:

8 $$W_{i}^{k} = \mathop \sum \limits_{j = 1}^{j = \left| V \right|} (e_{i,j}^{k} )\, where\,\, j \in \varGamma \left( i \right)$$

#### Definition of the weight summary of one measurement to two nodes

Let vertex *m* *∈ V*, and let edges from (*i, m*) and (*j, m*) exist, as shown in Fig. 3b. Equation (9) defines the summary values of measurements from vertex *m*, which is connected to *i* and *j*:

9 $$T_{i,j}^{k} = \mathop \sum \limits_{m = 1}^{m = \left| V \right|} \left( {e_{i,m}^{k} + e_{j,m}^{k} } \right)\;where\;\; m \in \varGamma \left( i \right) and m \in \varGamma \left( j \right)$$

#### Definition of structure similarity

Equation (10) defines the structure similarity of two vertices *i* and *j* as a vector:

10 $$Sim_{i,j} = \left\{ {S_{i,j}^{1} ,S_{i,j}^{2} ,S_{i,j}^{3} } \right\} = \frac{{\left\{ {T_{i,j}^{1} ,T_{i,j}^{2} ,T_{i,j}^{3} } \right\}}}{{\sqrt {W_{i}^{1} \cdot W_{j}^{1} + W_{i}^{2} \cdot W_{j}^{2} + W_{i}^{3} \cdot W_{j}^{3} } }}$$

#### Definition of the threshold neighbor

If two nodes can be clustered together based on measurement *k*, their structure similarity value $S_{i,j}^{k}$ must be greater than the preset threshold $\varepsilon^{k}$ to filter out noise. Equation (11) defines neighbors with qualified similarity structure values. The parameter $\varepsilon^{k}$ could be estimated via training. Thus, we invited users to perform the card-sorting task, which is described in section “Card-sorting task”, to create manual friend clusters. We also used different combinations of $\varepsilon^{k}$ to run our system. One of the combinations could produce the highest *F*_1_ score, as described in section “Performance evaluation”; this combination of $\varepsilon^{k}$ would thus be used in the mCAF algorithm:

11 $$N_{{\varepsilon^{k} }} \left( i \right) = \left\{ {j | j \in \varGamma \left( i \right) \wedge S_{i,j}^{k} \ge \varepsilon^{k} } \right\}\quad where\quad k = 1\;to\;3$$

Table 1 shows the pseudo code of the proposed mCAF algorithm. Initially, we set each vertex to be unclassified. We then calculate the values {${\text{e}}_{{{\text{i}},{\text{j}}}}^{1}$, ${\text{e}}_{{{\text{i}},{\text{j}}}}^{2}$, ${\text{e}}_{{{\text{i}},{\text{j}}}}^{3}$} and {${\text{S}}_{{{\text{i}},{\text{j}}}}^{1}$, ${\text{S}}_{{{\text{i}}, {\text{j}}}}^{2}$, ${\text{S}}_{{{\text{i}},{\text{j}}}}^{3}$}. STEP 1.1 attempts to determine the best measurement for clustering for each vertex. We then exam each *j*$\in \varGamma \left( i \right)$: if the largest value of { ${\text{S}}_{{{\text{i}},{\text{j}}}}^{\text{k}} \times\upmu^{\text{k}}$ } for *k* = 1…3 is { ${\text{S}}_{{{\text{i}},{\text{j}}}}^{\text{x}} \times\upmu^{\text{x}}$ }, then *count*^*x*^ = *count*^*x*^ + 1. Then, we determine the largest value in *count*^1^, *count*^2^, and *count*^3^. Assuming that the largest value is *count*^2^, we use measurement 2 for clustering with other vertices for *i*. We then store the value as *k*_*max*_(*i*) = 2, as shown in STEP 1.2. If we cannot determine the largest value from *count*^*x*^, vertex i will be tagged as *uncertainV*.

**Table 1:**
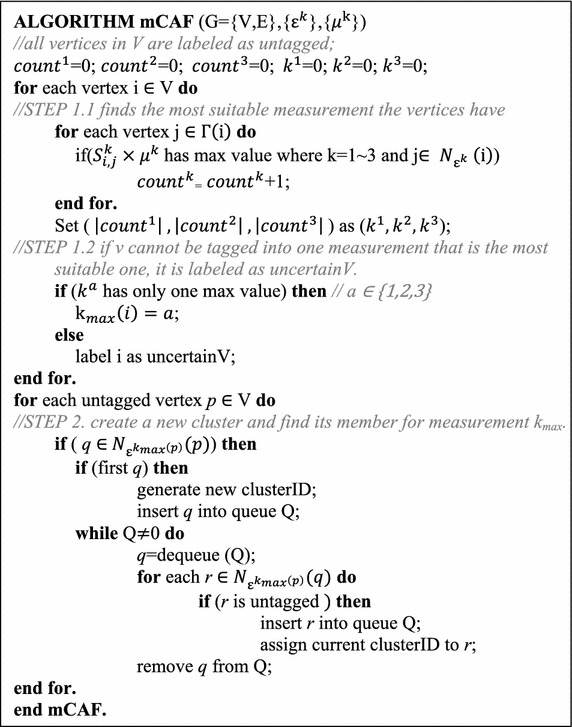
Pseudo code of the mCAF algorithm

In STEP 2, the mCAF algorithm will choose a random vertex *p* as the starting point and check each $q \in {\text{N}}_{{\upvarepsilon^{{{\text{k}}_{ \hbox{max} } \left( {\text{p}} \right)}} }} \left( p \right)$. If *q* is not tagged as any *clusterID*, *q* will be clustered together with p and tagged as a new *clusterID*. We will continue checking all untagged vertices in $N_{{\upvarepsilon^{{k_{max} \left( p \right)}} }} \left( q \right)$ and tag them as *clusterID* until no more vertices can be identified. We then choose another untagged vertex as a new starting point and perform the process again until all vertices in the graph are tagged.

In this step, we use the card-sorting method (Kelley et al. 2011), which requires subjects to manually cluster all of their Facebook friends. We prepare the same number of cards as that of the subject’s Facebook friends, where each card contains the Facebook profile picture and name of each friend. The subject sequentially places the paper cards on a table and stacks the cards that are classified in the same category. After completion, the subject provides each group with a name.

### Performance evaluation

We used three methods to quantify the performance of the proposed method: precision-recall, similarity, and improved ratio. The term “precision” represents the accuracy rate of the clustered friends who are clustered into the correct groups, while the term “recall” represents the accuracy rate of all friends who are clustered into correct groups. The F_1_ score is used to measure the combination of precision and recall to avoid biases in either recall or precision. First, we used the concepts of precision and recall to compare the subjects’ clustering using manual card-sorting, SCAN and mCAF using Eqs. (12) and (13), where *U* represents the number of friends classified by the subject (the ‘other’ category is excluded here), *A* represents the number of friends classified by the algorithm (*uncertainV* and other unclassified people are excluded here), and $U \cap A$ represents the number of friends in the same group in *U* and *A*. Then, we use Eq. (14) to compute the *F*_1_ scores:

12 $$Precision = \frac{U \cap A}{A}$$

13 $$Recall = \frac{U \cap A}{U}$$

14 $$F_{1} = \frac{2 \times Precision \times Recall}{Precision + Recall}$$

Second, we compared the similarities of groups between the subjects’ manual clustering and the SCAN algorithm’s and mCAF algorithm’s automatic clustering. We use Eq. (1) to perform these similarity calculations. Third, we use the average number of the similarity between mCAF and SCAN to calculate the improved ratio using Eq. (15):

15 $$improved\_ratio = \frac{{Average\left( {Value_{mCAF} } \right) - Average\left( {Value_{SCAN} } \right)}}{{Average\left( {Value_{SCAN} } \right)}}$$

### User adjustment

If more than two measurements are found to have the same or similar scores at this stage, such as the *uncertainV* node shown in Fig. 1, we consider this type of person to be a hub (Xu et al. 2007) that connects more than one cluster. Thus, because there is uncertainty in the group that the friend belongs to, we set the friend as an uncertain vertex. These people are identified during this step for manual clustering by the subject to make this application more flexible.

## Experiments

Facebook is currently the most widely used social networking service. More than one billion people use it every day; thus, we chose Facebook as our experimental data source. Graph API (version 2.0, 2014) is easy to understand and simple to use; Graph API was launched by Facebook. We used the Facebook Graph API to collect personal information from Facebook with the users’ consent. We retrieved different information based on a set of measurements, which is shown in Table 2.

**Table 2:** Corresponding measurements of items retrieved by the Facebook Graph API

Measurement	Features
Social circles	Mutual friends and article id in Facebook groups
Regions	Location of hometown and current residence
Organizations	Work and education
Tie strength	Photos, name tags, location tags, articles, groups, mutual friend lists, etc.

As shown in Table 3, there were a total of 20 subjects used in this experiment. The total number of their Facebook friends ranged from 79 to 837.

**Table 3:** Subject ID numbers and each subject’s number of Facebook friends

Subject ID	Number of Facebook friends	Subject ID	Number of Facebook friends
1	502	11	186
2	391	12	172
3	465	13	415
4	449	14	592
5	256	15	460
6	333	16	366
7	404	17	437
8	837	18	217
9	79	19	200
10	493	20	403

**Fig. 4:**
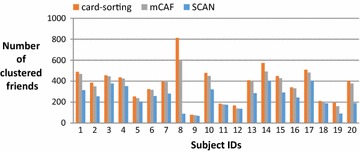
Number of clustered friends produced by the card-sorting, mCAF and SCAN methods

**Fig. 5:**
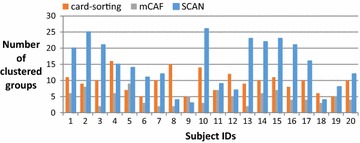
Number of clustered groups produced by the card-sorting, mCAF and SCAN methods

**Fig. 6:**
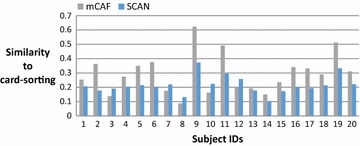
Similarities of the mCAF and SCAN methods to the card-sorting method

**Fig. 7:**
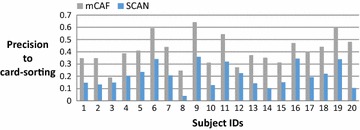
Precisions of the mCAF and SCAN methods with respect to the card-sorting method

**Fig. 8:**
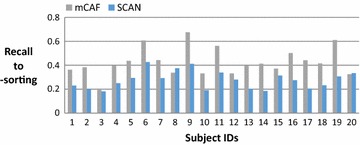
Recalls of the mCAF and SCAN method with respect to the card-sorting method

**Fig. 9:**
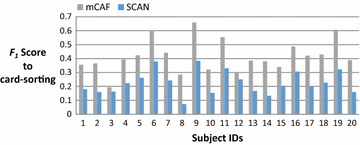
F_1_ scores of the mCAF and SCAN methods with respect to the card-sorting method

On average, the subjects in this study have 383 Facebook friends; thus, most are reluctant to manually cluster their friends and consider the task of clustering troublesome when we request them to do the card-sorting. Figure 4 shows the number of friends of these 20 subjects, and Fig. 5 shows the number of groups clustered by the card-sorting task, the (Kelley et al. 2011) based on SCAN algorithm (named SCAN in the following), and the proposed mCAF algorithm. In this figure, the SCAN algorithm is shown to cluster friends into more groups than either the manual card-sorting method or the mCAF algorithm. Figure 6 shows the similarity of the three methods, and Figs. 7, 8, and 9 show the precision, recall and *F*_1_ score values of the different methods.

As shown in Table 4, the card-sorting, SCAN and mCAF methods are compared by calculating the average precision, recall, *F*_1_ score and similarity. The improved ratios between mCAF and SCAN are 35.8 % in similarity and 84.9 % in *F*_1_ score.

**Table 4:** Average precision-recall and *F*_1_ scores of the mCAF and SCAN methods for 20 subjects

Method	Average precision (%)	Average recall (%)	Average *F*_1_ scores (%)	Average similarity (%)
SCAN	20.4	27.6	22.5	21.5
mCAF	40.8	42.7	41.6	29.2

## Conclusions

In this paper we proposed a new algorithm named mCAF which uses the concept of multi-dimensional relationships between different objects. We define these relationships as behaviors and connections in a social network system using friends as objects.

There are two primary contributions in this paper. The first contribution is the definition of four measurements that can be used to cluster friends in online social network systems: social circles, regions, organizations, and tie strength. We obtained specific information from Facebook and quantified this information into certain parameter values for calculation and comparison. We then used these values to define the distance between friends.

The second contribution of this study is a clustering algorithm called mCAF that can automatically cluster friends. The proposed mCAF algorithm considers both the network structure and the concept of an un-directed, weighted graph. Friends who have roles (e.g., a hub) are identified for manual clustering only, which lowers the chance of misjudgment by the algorithm. Based on our experimental results, the improved ratios between mCAF and SCAN are 35.8 % in similarity and 84.9 % in *F*_1_ score.
